# Semi-Automated Retrospective Surveillance of Surgical Site Infections (sAReS-SSI) – using hospital routine data for SSI tracking

**DOI:** 10.1186/s13756-026-01818-4

**Published:** 2026-08-31

**Authors:** Dominik Sons, Eva Bernauer, Alexander Schmidt, Meike Neuwirth, Robin Otchwemah, Heide Niesalla, Manuel Heurich, Frauke Mattner, Christoph Senges

**Affiliations:** 1 Institute of Hygiene, Cologne Merheim Medical Centre, University Hospital of Witten/Herdecke, Cologne, Germany; 2 Division of Hygiene and Environmental Medicine, Department of Human Medicine, Faculty of Health, Witten/Herdecke University, Witten, Germany; 3 BinDoc GmbH, Tübingen, Germany; 4 Central Department of Hygiene and Environmental Medicine, University Hospital Essen, University of Duisburg-Essen, Essen, Germany; 5 HARTMANN SCIENCE CENTER, BODE Chemie GmbH – a company of the HARTMANN GROUP, Hamburg, Germany

**Keywords:** Surgical site infection, Healthcare-associated infection, Infection surveillance, Semi-automated algorithm

## Abstract

**Background:**

Surgical site infections (SSIs) place a significant burden on healthcare systems worldwide. Although surveillance is crucial for obtaining accurate data and developing effective prevention strategies, there are still significant gaps due to the frequent reliance on manual and resource-intensive processes. To gain efficient, in-depth insight, we developed an algorithm for semi-Automated Retrospective Surveillance of Surgical Site Infections (sAReS-SSI), which retrospectively identifies SSIs using existing routine hospital data.

**Methods:**

sAReS-SSI adopts a patient-centric approach to data analysis, using an ICD-10 and OPS catalogue to detect *in-house* SSIs through temporal linkage to prior *in-house* surgeries, refining the NWIF algorithm of the German Institute for Quality and Transparency in Healthcare. sAReS-SSI was evaluated against SSIs that were validated through bedside surveillance in three orthopaedic and trauma surgery wards, as published in the HygArzt study. Its performance was also evaluated in comparison with a reconstructed NWIF algorithm, and contextualised using OP-KISS surveillance reports from the German National Reference Center.

**Results:**

sAReS-SSI correctly identified 61 of 65 *in-house* SSIs published in the HygArzt study. Re-analysis of initially false-positive cases revealed 10 additional true *in-house* SSIs not captured in HygArzt, yielding a sensitivity of 94.7%, specificity of 94.3%, positive predictive value of 35.9%, and negative predictive value of 99.8%. sAReS-SSI accurately captured infection dynamics in pre- and post-intervention periods of the HygArzt study, correctly identified problematic surgery types, and demonstrated that relying solely on NWIF or OP-KISS might miss a portion of potential SSI events.

**Conclusions:**

Automating the analysis of routine information for the detection of SSIs offers opportunities to save resources and enables efficient, large-scale analysis. By narrowing down potential SSI cases, this approach reduces the workload for manual surveillance by hygiene experts. In our cohort sAReS-SSI flagged potential cases and highlighted how routine-data-based case finding may extend OP-KISS surveillance by including additional non-indicator-surgeries.

**Supplementary Information:**

The online version contains supplementary material available at https://doi.org/10.1186/s13756-026-01818-4.

## Background

Healthcare-associated infections (HAIs), especially surgical site infections (SSIs), place a considerable burden on healthcare systems worldwide. In the European Union (EU) and European Economic Area (EEA), between 3% and 14% of hospitalized patients are affected by at least one HAI [1]. In Germany, the prevalence was up to 4.2% in hospitalized patients [1]. In addition, increasing rates of antimicrobial resistance are aggravating the situation [2–4]. At just over 16%, SSIs are among the three most common types of HAIs in the EU/EEA [1]. Surgical patients are often particularly susceptible to SSIs, which contribute significantly to morbidity, mortality, and the burden on the healthcare system [5]. Patients contracting an SSI have an average hospital stay that is 13 days longer (range: 4–24) [6–10] and a 2–11 times higher mortality risk [11–13]. However, not only patients, but also healthcare workers (HCWs) are affected by the impact of HAIs, which significantly increase their workload [14, 15]. The financial impact of SSIs on the healthcare system is also immense [7, 9, 16–24]. Additional healthcare costs of SSIs per infected patient ranged from €926 to €65,114 in Germany in 2016 [8], while individual hospitals face opportunity costs exceeding €1,000 per preventable case [25]. Furthermore, an analysis of German hospital reimbursement data (diagnosis-related groups; DRG) revealed a median underfunding of SSIs of around €1,500 per patient [26].

Preventing SSIs requires an understanding of their occurrence as well as effective prevention strategies. Surveillance of HAIs is crucial for obtaining accurate data on case numbers, locations, and the effect of interventions. However, significant gaps in surveillance remain a persistent challenge, especially as approaches rely heavily on manual processes. Further, much knowledge about HAI and SSI rates, mortality, and the success of interventions is based on data from research institutions like tertiary care university hospitals, potentially leading to bias, as significant resource gaps exist between academically led tertiary healthcare providers and smaller secondary care hospitals. Unlike smaller hospitals, research institutions have highly trained hygiene experts, high-quality data, and the necessary resources for interventions and advanced surveillance systems. Without comprehensive surveillance, however, it remains difficult to effectively identify and address the causes of SSIs. Automated and semi-automated surveillance, which is currently used predominantly in research institutions, might support high-quality surveillance while saving resources [27–29].

One widely used surveillance system in Germany is KISS (Krankenhaus-Infektions-Surveillance-System; hospital infection surveillance system) and its SSI-specific module, OP-KISS [30, 31], for voluntary surveillance of selected surgeries. Since this surveying is mostly done manually, it is often viewed as time-consuming, requiring many infection prevention and control (IPC) resources [32]. Automated approaches, on the other hand, present distinct challenges, as they require specialized IT resources [27] and often face a diversity of hospital IT infrastructures and software solutions. This makes automated, structured surveillance difficult, which requires consistent data structures [32]. Another challenge lies in distinguishing between *in-house* cases, defined as infections acquired during a procedure within the same hospital stay, and *external* cases, referring to infections resulting from accidents or treatments in other hospitals. In principle, retrospective SSI surveillance using standardized data is feasible, as demonstrated by the 2021 BARMER report [10]. It involved hospital routine data, as defined in Section 21 of the Hospital Remuneration Act [33], from one health insurance company, and used ICD-10 codes (International Classification of Diseases, 10th edition) to identify potential SSI cases. Another example, relying on ICD and OPS (Operationen- und Prozedurenschlüssel; surgery and procedure codes) codes, is the NWIF algorithm (Nosokomiale WundInFektionen; case-related healthcare-associated wound infection), which was used within the German quality assurance framework to identify potential SSI cases. The NWIF algorithm was part of a mandatory, cross-institutional and -sectoral SSI surveillance trial, coordinated by the IQTIG (Institut für Qualitätssicherung und Transparenz im Gesundheitswesen; Institute for Quality Assurance and Transparency in Healthcare), until 2026, using ICD-10 and OPS-codes (OPS codes are the German equivalent to the US ICD-10-PCS) [34]. So far, neither of the automated approaches has been validated, and the NWIF trial relied heavily on manual confirmation of SSI candidates.

In this study, we present the ‘semi-Automated Retrospective Surveillance of Surgical Site Infections’ (sAReS-SSI) algorithm for the retrospective identification of *in-house* SSI candidates. The algorithm leverages standardized hospital routine data, such as ICD-10 and OPS codes, including timestamps, to detect SSIs in a scalable and more resource-efficient manner. Building on the NWIF algorithm, we developed and validated sAReS-SSI using data from the HygArzt study [35], which was conducted in three orthopaedics and trauma surgery wards and in which SSIs were identified through prospective surveillance and manually confirmed by an IPC team. We applied the sAReS-SSI algorithm to the study population and compared identified SSI cases with those detected in the HygArzt study, re-examining any discrepant cases. Subsequently, we compared the performance of sAReS-SSI and the reconstructed NWIF algorithm at the patient level and identified surgeries and SSIs included in the standardized OP-KISS surveillance.

## Methods

### Study design

Our study was a retrospective secondary data analysis based on the dataset from the HygArzt pilot study, a prospective interventional cohort study conducted from March 2018 to June 2019 on three orthopaedic and trauma surgery wards at one university hospital [35, 36]. The HygArzt study comprised a 6-month pre-intervention phase (1 March 2018 to 31 August 2018), followed by a 4-month intervention phase, and a 6-month post-intervention phase (1 January 2019 to 30 June 2019). The intervention involved the implementation of an infection prevention bundle by an infection prevention link physician, who served as a liaison between the IPC team and the clinical department, with the aim of reducing infection rates [35]. In the HygArzt study, SSIs were identified through prospective surveillance of all consecutive patients with surgical procedures during the study periods, whereas our semi-automated approach relied retrospectively on standardized hospital routine data. Routine data contains diagnoses as ICD-10 codes and procedures as OPS codes with dates, as well as admission and discharge dates, and can be extracted from claims data. As patient records differ from hospital routine data, our newly generated dataset differed slightly from the original HygArzt dataset. This served as the basis for applying sAReS-SSI to identify *in-house* SSI cases. We defined SSIs as ‘*in-house*’ if the infection was acquired following surgery at the hospitals participating in the HygArzt study. SSIs classified as ‘*external*’ comprised both community-acquired infections and SSIs acquired at hospitals not involved in the HygArzt study. Whenever discrepancies occurred between our results and those of the HygArzt study, we manually re-analyzed the patient records. Finally, we compared the performance of sAReS-SSI with that of a reconstructed NWIF algorithm and with *in-house* OP-KISS surveillance results.

### HygArzt (Published) – The original dataset from the HygArzt study

From a total of 2,643 patients, 2,480 were included and prospectively surveilled for the development of an HAI in the HygArzt study [36]. Subsequently, this dataset is referred to as HygArzt (published). IPC professionals identified 70 SSIs, of which 44 occurred in the pre-intervention phase and 26 in the post-intervention phase [35]. The study design and methodological approach, including inclusion and exclusion criteria, have been described previously [36]. In short, all patients undergoing orthopedic-traumatological surgery and with inpatient admission to one of the associated normal/general wards were included. Infection surveillance was limited to 90 days after the last surgery on the respective body site, according to OP-KISS definitions [30]. Infections brought along, acquired in an intensive care unit, or that developed in less than 48 h after admission were excluded. Data from patients leaving the hospital against medical advice were also excluded [36].

### HygArzt (Routine data) – Deviation between the original HygArzt dataset and the sAReS-SSI dataset

We extracted data for all patients included in HygArzt from hospital routine data pursuant to Section 21 of the Hospital Remuneration Act, creating the HygArzt (routine data) dataset [33]. This dataset includes case-related information transmitted to health insurance funds for billing medical treatments. It contains admission and discharge dates, diagnoses according to the German ICD-10 classification (G-ICD-10, subsequently described as ICD-10), and surgeries and procedures according to the OPS system (i.e. non-pharmacological treatments) [37–39].

**Fig. 1:**
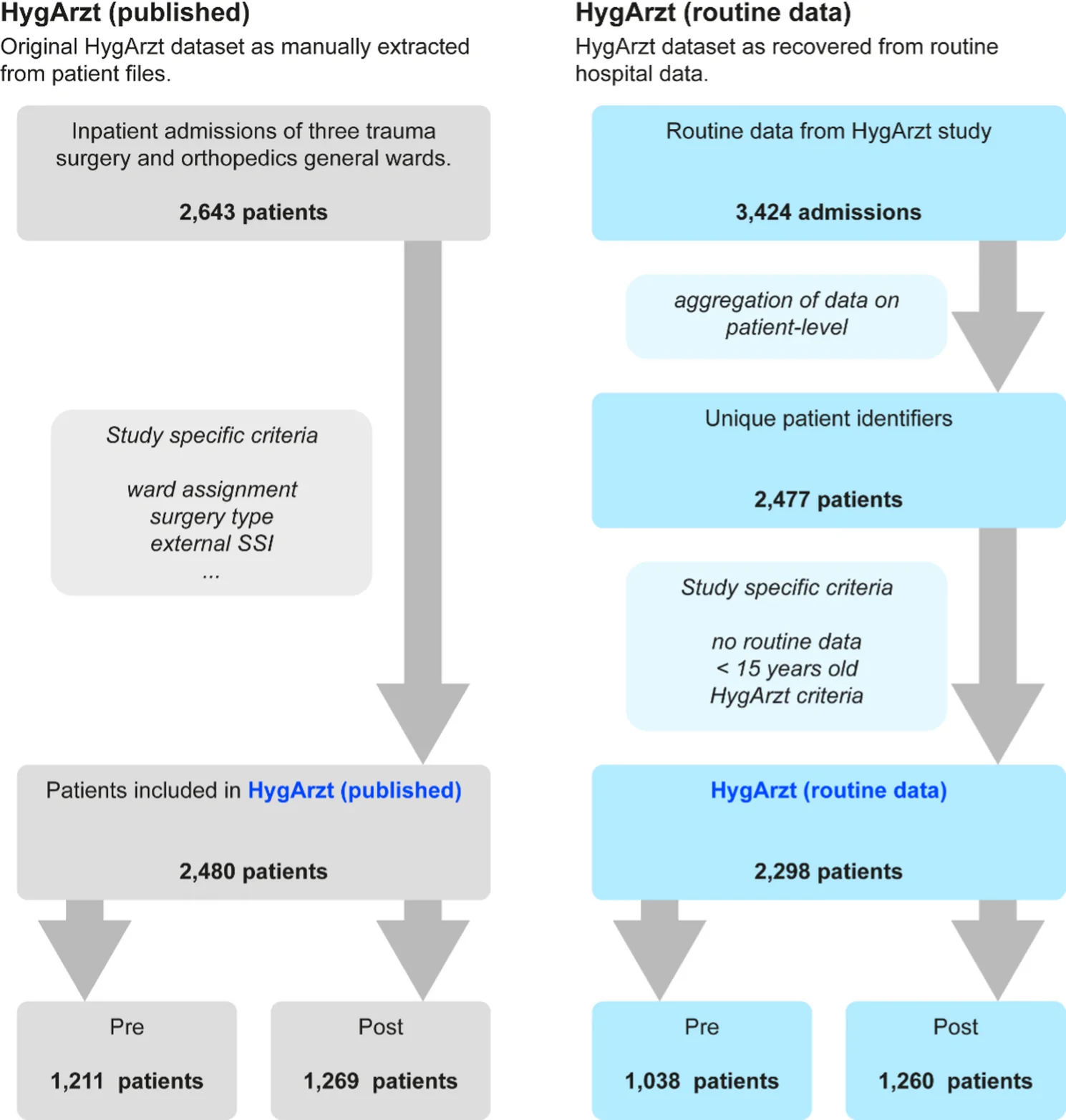
Processing of the datasets based on patient records and hospital routine data. The number of patients in the pre- and post-intervention phases of the HygArzt project, found by manual analysis of patient records (HygArzt (published)) and automated detection in hospital routine data (HygArzt (routine data)). Final datasets are highlighted in bright blue. SSI, surgical site infection

The original dataset from HygArzt initially included 3,424 admissions, which, after data processing, corresponded to 2,477 unique patient IDs (Fig. 1). Of these, 20 patients did not appear in the hospital routine data, resulting in a preliminary dataset of 2,457 patients. In our study, we excluded patients younger than 15–20 years (only age brackets from hospital routine data available). We then applied further requirements from the HygArzt study: The (initial) surgery had to have taken place within the pre- and post-interventional phases, and the patients had to have been inpatients on one of the three included wards at some point during the pre- or post-interventional phase. These criteria reduced our dataset to a final number of 2,298 patients (pre-intervention: 1,038; post-intervention: 1,260; Fig. 1; Table 1), hereafter referred to as HygArzt (routine data), while the HygArzt (published) dataset included 2,480 patients (pre-intervention phase: 1,211; post-intervention phase: 1,269) [35].

**Table 1:** Comparison of the number of patients, hospital stays, and surgeries found by manual analysis of patient records (HygArzt (published)) and automated detection in hospital routine data (HygArzt (routine data))

	HygArzt (routine data)		HygArzt (published)		Difference routine data vs. published	
	pre- intervention	post- intervention	pre- intervention	post- intervention	pre- intervention	post- intervention
	**N**	**N**	**N**	**N**	**%**	**%**
Patients	1,038	1,260	1,211	1,269	-14.2%	-0.7%
Hospital stays	1,200	1,535	1,413	1,401	-15.1%	+ 9.6%
Surgeries*	1,330	1,694	1,430	1,583	-7%	+ 7%

*Surgeries included exclusively OPS codes starting with 5- and were performed on different dates when the same patient was involved. OPS, Operationen- und Prozedurenschlüssel; surgery and procedure codes

### Reconstructed NWIF

To establish a methodological baseline and enable comparison with an accepted reference standard, we reconstructed the NWIF algorithm of the IQTIG using publicly available information [40, 41]. In this context, ‘reconstructed’ refers to the recreation of NWIF retaining its original detection mechanisms, while shifting from a case-level to a patient-level approach. The NWIF mechanism consists of two components. First, it defines a set of diagnostic and procedural codes indicating a potential SSI on the case level, accompanied by exclusion criteria such as immune deficiency, diverticulitis with perforation, burns, polytrauma, amputation, or chemotherapy (full list provided in [40]). Second, NWIF requires a subsequent manual post-recording of document timestamps, pathogen detection, the link between surgery and SSI, and the SSI-classification as *in-house* or *external*. In routine practice, cases flagged by the NWIF algorithm as SSI candidates must be manually re-assessed by physicians.

### sAReS-SSI algorithm

**Fig. 2:**
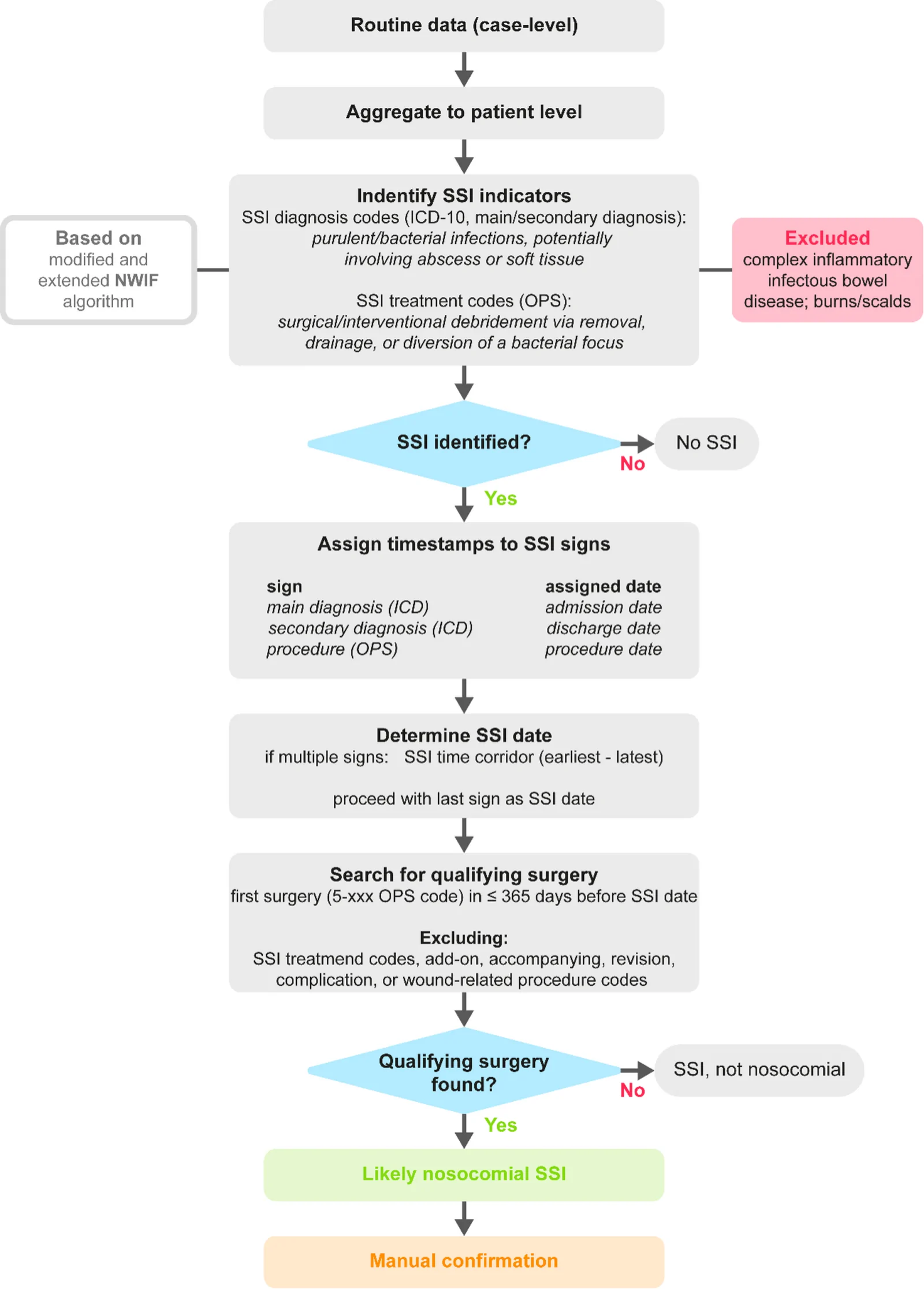
Flowchart of the sAReS-SSI algorithm. Dataflow in sAReS-SSI for the automated identification of likely *in-house* SSI candidates in hospital routine data. Hospital routine data is aggregated from case level to patient level. Putative SSIs are identified based on SSI diagnosis or treatment and subsequently filtered for signs of *in-house*, nosocomial SSIs, resulting in a list of SSI candidates. Subsequently, the candidates were verified manually. An SSI is considered to be *in-house* if it is acquired during surgery within the same hospital. ICD-10, International Classification of Diseases 10th edition; NWIF, module for the case-related documentation of wound infections; OPS, Operationen- und Prozedurenschlüssel - surgery and procedure codes; SSI, surgical site infection

Building on NWIF, we developed the sAReS-SSI algorithm (Fig. 2), aiming to reduce the number of ‘SSI candidates’ to ‘likely *in-house* SSIs’ to minimize the workload for manual review. To capture multiple hospital stays per patient and reflect the delayed onset of SSIs, we aggregated case-level data from 2018 to 2020 (discharge year) at the patient level. For identification of SSI candidates, we used NWIF ICD-10 codes indicating infection, inflammation, abscess, etc., some requiring confirmed pathogen detection coded in claims data as the main or secondary diagnosis, as well as NWIF OPS codes with timestamps signaling SSI treatment (e.g., wound toilet, debridement, excision of diseased tissue). Unlike NWIF, we did not require the combination of ICD-10 and OPS codes. We also relaxed the exclusion criteria (e.g., immune deficiency, polytrauma, chemotherapy) and added missing ICD-10 codes. To reconstruct the temporal sequence of SSI treatment, the main diagnosis was taken as the primary reason for hospital admission, using the admission date as the reference. As secondary diagnoses lack timestamps and can occur at any point during the stay, we conservatively used the discharge date as a reference point. OPS codes include timestamps, allowing precise identification of interventions. Based on this information, we extracted the first and last recorded signs of an SSI for each patient with associated timestamps, requiring the first sign to occur no later than one year after the end of the study period (June 30, 2020). If a patient was admitted with an SSI ICD-10 code as the main diagnosis for the first stay, this infection was classified as an ‘*external*’, not an ‘*in-house’* SSI. However, if an SSI ICD-10 code appeared as the main diagnosis in a subsequent stay following an *in-house* surgery, it was counted as an *in-house* SSI. We also required that the initial surgery (first surgery within the pre- or post-intervention phase) occurred before the SSI. As some of the SSI signs were ambiguous, the last recorded sign was used as the reference point. Further criteria for the initial surgery were that it occurred within 365 days before the SSI and that the OPS code started with 5-, indicating a surgery [39]. We excluded OPS codes for initial surgeries starting with 5- if they indicated the treatment of an SSI, such as surgical wound debridement or temporary soft tissue coverage, or if they clearly did not classify as primary surgery according to a pre-defined list of codes. Acknowledging that perfect identification of *in-house* SSIs is not possible, ambiguous cases were retained for subsequent manual verification.

### Data analysis and statistics

To examine the study population, the characteristics of the participants in the pre- and post-intervention phases were compared to determine whether they matched the characteristics of the original HygArzt study concerning gender, mean age, and comorbidities [35]. While the HygArzt study provided the American Society of Anesthesiologists (ASA) score [42], we calculated the Quan version of the Charlson Comorbidity Index (CCI) [43], which roughly estimates the mortality risk for 12 underlying diseases (originally 17; 5 of these with a factor of 0; therefore, 12 in the modified score).

In addition to the patient characteristics of the HygArzt study, the sample was further characterized by the number of surgeries (at any time point and with different unique dates), the ten most frequent main diagnoses, the ten most frequent initial procedures, and the ten most frequent OP-KISS surgeries [31] according to the current OP-KISS classification (2025) [44].

Continuous variables were presented as the mean (standard deviation, SD) and binary or categorical variables as n (%) of positive cases per group. Statistical significance was assessed using Welch’s test for continuous variables and Pearson’s chi-squared test or Fisher’s exact test for binary or categorical variables. To compare SSI rates between our sAReS-SSI analysis and the HygArzt study [35], infection rates for the pre- and post-intervention phases were presented, and the relative risk (RR) with 95% confidence intervals (CIs) was calculated. To evaluate the predictive performance of sAReS-SSI compared to the published HygArzt classifications, confusion matrices were created, and sensitivity, specificity, positive predictive value (PPV), negative predictive value (NPV), and F1 scores were calculated. As the harmonic mean of PPV and sensitivity, the F1 score summarises both performance parameters into a single value. Sensitivity and specificity were compared between paired algorithms using McNemar’s test to account for correlated results within the same patient cohort. PPV and NPV were compared using two-sided z-tests for proportions. 95% CIs for all performance metrics were calculated using the Wilson score method for binomial proportions. Putative false-positive cases that sAReS-SSI predicted to have an SSI, but which were not classified as such in HygArzt, were reviewed manually and reclassified if necessary. The confusion matrix and all quality metrics were then recalculated.

Non-SSI and SSI cases were compared with respect to patient characteristics for the pre- and post-intervention phases. Similar to Neuwirth et al. [35], infection rates were reported by body region; however, our classification was based on OP-KISS procedures as indicators for body regions rather than manual classification, as used in the HygArzt study. To identify these OP-KISS body regions, the SSI cases were traced back until the first KISS procedure/category prior to the SSI was found for the patient within the observation period. If no such KISS procedure was found, the patient was consequently not labelled with a nosocomial SSI. Note that the qualifying procedure from the original sAReS-SSI and this “KISS-adapted” sAReS-SSI may diverge. All statistical analyses were performed using Python 3.12.12, employing pandas (version ≥ 2.1.0) for data handling, NumPy (version ≥ 1.26.0) for numerical operations, and SciPy (version ≥ 1.11.0) for statistical testing.

## Results

### Evaluation of sAReS-SSI using SSI candidates identified in the HygArzt study

Of the 70 SSIs originally identified in the HygArzt (published) dataset, 65 were available in the HygArzt (routine data) dataset and were eligible for analysis using sAReS-SSI. The remaining five SSI cases were not included in the inpatient hospital routine data and were therefore not assessable. Among the evaluable 65 SSIs, sAReS-SSI correctly identified 61 and missed four. One of the four unidentified cases was overlooked because the SSI occurred during an outpatient visit and was only documented in the discharge letters based on the physician’s diagnosis. The second case was correctly assigned to an *external* SSI; however, a subsequent *in-house* SSI following the initial episode was not detected by sAReS-SSI. More precisely, the definition of a new wound infection on the same body region required a 14-day healing interval and the identification of a different pathogen – criteria that could not be adequately captured using ICD-10 codes alone [31]. The third case was not flagged by sAReS-SSI because an infection had already been coded during a previous hospitalization, so the case was classified as *external*. The fourth patient underwent an *in-house* outpatient procedure that was not recorded in our hospital routine dataset, which is why the infection during the subsequent admission was classified as *external* as well. A direct comparison of sAReS-SSI and the original HygArzt data yielded a sensitivity of 93.8%, a specificity of 93.9%, a PPV of 30.8%, an NPV of 99.8% and an F1 score of 46.4% (sAReS-SSI (initial dataset); Table 2).

**Table 2:** Comparison of SSI identification performance of sAReS-SSI and NWIF algorithms

Algorithm (routine data)		*N* = 2,298		Sensitivity	Specificity	PPV	NPV
		SSI		%	%	%	%
		True	False	[95% CI] *p*-value	[95% CI] *p*-value	[95% CI] *p*-value	[95% CI] *p*-value
sAReS-SSI (initial)	SSI positive	61	137	93.8 [85.2–97.6]	93.9 [92.8–94.8]	30.8 [24.8–37.6]	99.8 [99.5–99.9]
	SSI negative	4	2096				
	compared with NWIF (initial)			*p* = 0.035	*p* < 0.001	*p* = 0.002	*P* = 0.020
sAReS-SSI (revised)	SSI positive	71	127	94.7 [87.0-97.9]	94.3 [93.2–95.1]	35.9 [29.5–42.8]	99.8 [99.5–99.9]
	SSI negative	4	2096				
	compared with NWIF (revised)			*p* = 0.013	*p* < 0.001	*p* < 0.001	*p* = 0.006
sAReS-SSI (revised with pathogen code)	SSI positive	68	84	90.7 [81.9–95.4]	96.2 [95.4–96.9]	44.7 [37.0-52.7]	99.7 [99.3–99.8]
	SSI negative	7	2139				
NWIF* (initial)	SSI positive	52	211	80.0 [68.7–87.9]	90.6 [89.3–91.7]	19.8 [15.4–25.0]	99.4 [98.9–99.6]
	SSI negative	13	2022				
NWIF* (revised)	SSI positive	60	203	80.0 [69.6–87.5]	90.9 [89.6–92.0]	22.8 [18.2–28.3]	99.3 [98.8–99.6]
	SSI negative	15	2020				

Evaluation of sAReS-SSI using the patient cohort from the HygArzt study compared to the reconstructed NWIF. Both algorithms use the initial HygArzt (routine) dataset with SSI classifications from the HygArzt study, as well as a revised dataset that includes SSI cases missed during the HygArzt study. The following performance parameters were calculated for each statistical test: sensitivity (true positive rate), specificity (true negative rate), PPV, and NPV. Confidence intervals for all parameters and both algorithms were calculated using the Wilson score method. Sensitivity and specificity were assessed with McNemar’s test for paired data. PPV and NPV were compared using two-sided z-tests for proportions. *Reconstructed NWIF algorithm based on publicly available information, modified for patient-level analysis. sAReS-SSI, semi-Automated Retrospective Surveillance of Surgical Site Infections; CI, confidence interval; NPV, negative predictive value; NWIF, module for the case-related documentation of wound infections; PPV, positive predictive value; SSI, surgical site infection

Overall, sAReS-SSI identified 198 potential SSI candidates. This included 61 patients already classified as SSI cases in the HygArzt study as well as 137 additional candidates (sAReS-SSI (initial dataset); Table 2). We initially classified those SSIs as presumably false positives. Manual re-analysis of those cases revealed 10 additional SSIs, increasing the total number of SSIs to 75, with 71 being identified by sAReS-SSI. Thus, 13.3% of SSIs had not been detected in the HygArzt study. Reasons why SSI cases were not detected in the HygArzt study included treatment approaches without microbiological sampling, conservative therapy with antibiotics instead of surgical revision, contradictions between physicians’ statements and OP-KISS definitions, and human error. In comparison, sAReS-SSI missed four (5.3%) of the 75 SSIs, findable in hospital routine data (sAReS-SSI (revised dataset); Table 2).

For both the initial and revised datasets, sAReS-SSI flagged fewer false positives than the reconstructed NWIF algorithm. While NWIF flagged 203 false positives as SSIs (NWIF (revised dataset); Table 2), sAReS-SSI flagged 127 false positives (sAReS-SSI (revised dataset); Table 2), reducing the workload for required subsequent manual analysis. Overall, sAReS-SSI overestimated the number of actual *in-house* SSIs by a factor of 2.64. Revision of the sAReS-SSI classification resulted in an improved sensitivity of 94.7%, specificity of 94.3%, PPV of 35.9%, and F1 score of 52.0%, while the NPV (99.8%) remained unchanged (sAReS-SSI (revised dataset); Table 2).

### False positive SSI flags by sAReS-SSI were ambiguous or complex cases

Among the cases falsely flagged as positive by sAReS-SSI and classified as negative after manual review were 19 *in-house* SSIs that did not meet all criteria of the original HygArzt study, as the infection was either due to a surgical procedure in another medical department, had developed in an intensive care unit, or had occurred outside the pre- or post-intervention phase. Had these cases also been taken into account, the PPV would have been 45.5%, the NPV 99.8%, the F1 score 62.0%, the sensitivity 95.7%, and the specificity 95.1%. While 16 cases were *external* SSIs, some cases involved complex procedures with both *external* and *in-house* components, where the initial surgery or first aid was performed *externally*, while subsequent, more specific treatment was provided *in-house*. Another 79 infectious cases were not counted as *in-house* SSIs, but their analysis was still informative. These cases included, for example, necrosis, severe accidents, and polytrauma with contaminated wounds, as well as cases without visual infection confirmation or pathogen detection. Adding pathogen detection as a requirement to our sAReS-SSI algorithm increases the PPV to 44.7%, specificity to 96.2%, and the F1-Score to 59.9%, while reducing sensitivity to 90.7% (Table 2).

### Comparison of patient and procedure characteristics – HygArzt (Published) vs. HygArzt (Routine data)

To ensure alignment of our findings derived from hospital routine data with those of the HygArzt study [35], which were based on intensive bedside surveillance and manual review of patient records, we also assessed the underlying patient and procedural characteristics and found them to be comparable (Table S1 and S2). The overall number of surgeries was also comparable (HygArzt (published) 3,013 vs. HygArzt (routine data) 3,024; Table 3).

**Table 3:** Comparison of infection rates in pre- and post-intervention phases (HygArzt vs. NWIF vs. sAReS-SSI)

Dataset	Pre-intervention phase			Post-intervention phase			RR [95% CI]	Pre vs. Post *p*-value
	SSIs	Surgeries	Infection rate	SSIs	Surgeries	Infection rate		
HygArzt (published)	44	1,430	3.1%	26	1,583	1.6%	0.54 [0.34–0.88]	0.010
HygArzt (routine data, initial)	41	1,330	3.1%	24	1,694	1.4%	0.46 [0.28–0.76]	0.002
HygArzt (routine data, revised*)	44	1,330	3.3%	31	1,694	1.8%	0.55 [0.35–0.87]	0.013
NWIF** (routine data, revised*)	126	1,330	9.5%	137	1,694	8.1%	0.85 [0.68–1.08]	0.19
sAReS-SSI (routine data, revised*)	99	1,330	7.4%	99	1,694	5.8%	0.79 [0.60–1.03]	0.088
sAReS-SSI (routine data, revised* + pathogen codes)	82	1,330	6.2%	70	1,694	4.1%	0.67 [0.49–0.91]	0.012

Infection rates for pre- and post-intervention phases, as reported in the HygArzt study (published), were calculated based on the sAReS-SSI and NWIF datasets (routine data) corresponding to the respective time periods. Patient numbers classified by the algorithms as positive for SSIs were related to the total number of surgeries performed in each phase. Statistical significance of differences observed between study phases due to the intervention is reported as p-values. *Including 10 SSIs that were missed in the original HygArzt study. **Reconstructed NWIF algorithm based on publicly available information, modified for patient-level analysis. sAReS-SSI, semi-Automated Retrospective Surveillance of Surgical Site Infections; CI, confidence interval; NWIF, module for the documentation of wound infections; RR, relative risk; SSI, surgical site infection

### Comparison of infection rates and relative risk (sAReS-SSI vs. HygArzt vs. NWIF)

sAReS-SSI led to an approximately 2.64-fold overestimation of SSIs. Thus, the sAReS-SSI-based infection rates in both study phases (pre-/post-intervention: 7.4%/5.8%) and the RR (0.79 [0.60–1.03]) were higher than in the published HygArzt study (pre-/post-intervention: 3.1%/1.6%; RR 0.54 [0.34–0.88] (Table 3). Yet, sAReS-SSI was able to capture the downward trend of SSI rates (Table 4), already evident at the end of the pre-intervention phase (Figure S1).

**Table 4:** OP-KISS procedures during the HygArzt study with SSIs predicted by sAReS-SSI

Level	Code	Pre-intervention phase			Post-intervention phase		
		No SSI	SSI	*p*-value	No SSI	SSI	*p*-value
Not_OP-KISS		459 (45.8%)			568 (46.5%)		
OP-KISS (all)		543 (54.2%)	36 (100.0%)		654 (53.5%)	38 (100.0%)	
Arthroscopic surgeries	ART	234 (23.4%)	7 (19.4%)	0.585	308 (25.2%)	7 (18.4%)	0.342
Hip replacement in osteoarthritis	HPRO_A	92 (9.2%)	7 (19.4%)	0.073	100 (8.2%)	5 (13.2%)	0.239
Knee replacement	KPRO	79 (7.9%)	5 (13.9%)	0.205	81 (6.6%)	4 (10.5%)	0.318
Surgery on the upper ankle joint	OSG	43 (4.3%)	9 (25.0%)	< 0.001	65 (5.3%)	6 (15.8%)	0.017
Reposition of proximal femur fracture	FPF_G	29 (2.9%)	0 (0.0%)	0.620	36 (2.9%)	2 (5.3%)	0.319
Hip replacement revision surgery	HPRO_R	16 (1.6%)	2 (5.6%)	0.126	13 (1.1%)	4 (10.5%)	0.001
Spinal fusion	SPONDY	13 (1.3%)	2 (5.6%)	0.093	24 (2.0%)	3 (7.9%)	0.045
Shoulder replacement	SPRO	12 (1.2%)	0 (0.0%)	1.000	10 (0.8%)	1 (2.6%)	0.287
Open reposition of proximal femur fracture	FPF_O	9 (0.9%)	1 (2.8%)	0.299	5 (0.4%)	2 (5.3%)	0.017
Knee replacement revision surgery	KPRO_R	8 (0.8%)	0 (0.0%)	1.000	6 (0.5%)	1 (2.6%)	0.193
Correction of a hallux valgus deformity, foot	HALLUX	4 (0.4%)	0 (0.0%)	1.000	2 (0.2%)	0 (0.0%)	1.000
Appendectomy	APPE	2 (0.2%)	0 (0.0%)	1.000			
Nephrectomy	NEPH	0 (0.0%)	1 (2.8%)	0.035			
Arterial reconstruction; lower extremity	GC_EXT	0 (0.0%)	1 (2.8%)	0.035	1 (0.1%)	2 (5.3%)	0.003
Cholecystectomy	CHOL	1 (0.1%)	0 (0.0%)	1.000			
Inguinal hernia	HERN	0 (0.0%)	1 (2.8%)	0.035	1 (0.1%)	0 (0.0%)	1.000
Craniotomy	KRAN	1 (0.1%)	0 (0.0%)	1.000	1 (0.1%)	0 (0.0%)	1.000
Vascular surgery, proce-dures on abdominal aorta	GC_ABD				1 (0.1%)	1 (2.6%)	0.059

Abbreviations in brackets represent procedure abbreviations of the OP-KISS module. p-values indicate significance of differences between “no SSI” and “SSI” per study phase. sAReS-SSI, semi-Automated Retrospective Surveillance of Surgical Site Infections; OP-KISS, operative module of hospital infection surveillance system; SSI, surgical site infection

### Comparison of sAReS-SSI with the OP-KISS standard

To investigate how sAReS-SSI could be used in conjunction with OP-KISS, we searched specifically for OP-KISS procedures. This meant that only a subset of all SSIs was identified, but it still provided a useful framework for categorizing surgeries by body regions. OP-KISS covered more than 50% of all surgeries (Table 4) but only around 37% of alleged SSIs (pre-/post-intervention: 36.4% (36 of 99)/38.4% (38 of 99)). In the pre-intervention phase, OP-KISS procedures most frequently associated with SSIs were surgeries on the upper ankle joint (*n* = 9; 25.0%; *p* < 0.001), followed by hip replacement in osteoarthritis (*n* = 7; 19.4%; *p* = 0.073), and arthroscopic surgeries (*n* = 7; 19.4%; *p* = 0.585). In the post-intervention phase, arthroscopic surgeries (*n* = 7; 18.4%; *p* = 0.342) ranked first among patients with an SSI, followed by surgeries on the upper ankle joint (*n* = 6; 15.8%; *p* = 0.017), and hip replacement in osteoarthritis (*n* = 5; 13.2%; *p* = 0.239) (Table 4). The original HygArzt study came to similar conclusions and also found surgeries on the lower extremities (hip and lower leg) to be associated with a higher risk of SSIs [35].

## Discussion

Surveillance of surgical site infections is a highly important, yet resource-intensive, process. Building on the IQTIG NWIF algorithm, our study shows that sAReS-SSI efficiently identifies potential *in-house* SSI candidates. The information obtained retrospectively from the hospital’s routine data proved to be comparable to the prospectively collected data based on patient records used in the HygArzt study. Using this approach, sAReS-SSI identified SSIs with a sensitivity of 94.7%, specificity of 94.3%, PPV of 35.9%, and NPV of 99.8%. When pathogen detection was additionally required, specificity increased by 2% points, and PPV increased to 44.7%, accompanied by a reduction in sensitivity to 90.7%. Although a PPV of approximately 40% may appear modest, it represents a strong performance in the context of a low-prevalence condition, as the underlying SSI rate in the studied population is approximately 3%. Importantly, while capturing most SSIs, our approach also reflected changes in SSI rates over time. Consequently, we were able to observe the effects of interventions, such as the hygiene measures implemented during the HygArzt intervention phase. However, as sAReS-SSI overestimates the absolute number of SSI cases, it cannot accurately estimate the magnitude of intervention effects or absolute SSI rates. Although manual analysis of patient records and active surveillance during the patient stay are considered the gold standard for SSI identification, this approach is not infallible due to the volume and variety of documents to be reviewed and the time required on wards. While the sAReS-SSI algorithm produces a considerable number of false positives, albeit fewer than the reconstructed NWIF algorithm, it still requires manual revision of flagged cases by a physician or the IPC team. Accordingly, sAReS-SSI should be considered a semi-automated SSI case-finding tool within the surveillance workflow rather than a fully automated diagnostic system. However, it can reduce the manual workload and support targeted reviews. Based on our experience, review of a flagged case typically required approximately 30 min, depending on case complexity and data availability. As only 198 of 2,298 patients required detailed review, the algorithm substantially reduced the number of records requiring manual assessment. Thus, we assume that using sAReS-SSI could contribute to striking a better balance between resource expenditure and insights gained. Because sAReS-SSI relies exclusively on routinely collected administrative hospital data, implementation and maintenance requirements are comparatively modest. Prior to deployment in new specialties or institutions, local validation is advisable to account for differences in coding practices and treatment procedures. Maintenance primarily consists of incorporating updates to coding and surveillance definitions.

The previously existing (semi-)automated analysis method in Germany, the NWIF algorithm, was discontinued by the end of 2025 and replaced by a module for sepsis surveillance [45, 46]. sAReS-SSI therefore represents an option for the subsequent surveillance of SSIs, characterized by higher specificity and sensitivity. The algorithm allows routine data to be analysed at flexible time intervals and can support hospital-wide screening for potential SSI cases based on routinely available administrative data. OP-KISS is the most important framework for manual analysis and standardized quality assurance, yet only for selected surgeries. By focusing on all surgical procedures with a 365-day follow-up window, sAReS-SSI might serve as a useful complement for OP-KISS surveillance, which, in contrast, covers significantly shorter observation periods (i.e., 30 or 90 days postoperatively, depending on the indicator procedure [47]). The 365-day observation period was chosen to maximise detection and can be adapted to shorter surveillance time frames if required. To our knowledge, no published studies have used a (semi-)automated algorithm for comprehensive, hospital-wide SSI screening across extended observation periods, retrospectively, using a manual validation study dataset [27, 48–50]. In principle, an active and prospective SSI surveillance approach using big data would require all relevant information sources to be integrated in order to meet CDC criteria, such as microbiological findings, wound parameters, or fever records. However, implementing such software remains challenging, as linking these heterogeneous data sources within hospital information systems is complex. Our approach represents a pragmatic alternative that can be implemented using routinely available administrative data while requiring substantially less integration effort than more comprehensive surveillance systems. In this context, we evaluated the performance of our algorithm in detail against the gold standard of active surveillance. Nevertheless, other authors agree with us that, at this time, automated surveillance approaches cannot and should not aim to eliminate human involvement entirely. Rather, their primary objective should be to significantly reduce the manual workload while improving standardisation and accuracy [27, 48–50]. Therefore, sAReS-SSI represents a promising and practical approach to identifying potential SSI hotspots in hospitals with multiple surgical departments and wards, enabling targeted allocation of hospital IPC expertise.

### Limitations

The main limitation of our study is the absence of external validation using data from an independent hospital, which limits the generalizability of our findings. Although sAReS-SSI operates on routinely collected administrative hospital data that are available in standard clinical practice, the present evaluation is a validation within a single-centre orthopaedic and trauma surgery cohort. Consequently, algorithm performance may differ across hospitals, specialties and coding practices. Validation in additional institutions and surgical disciplines is therefore warranted before broader implementation. sAReS-SSI is limited to information available within routine inpatient data. Therefore, it may not detect infections managed in other healthcare facilities or outpatient settings, infections documented exclusively in unstructured clinical documentation, or SSI episodes following outpatient procedures. In particular, superficial SSI cases may therefore be underrepresented. Additionally, data from active surveillance, patient records, and routine data are not perfectly congruent, leading to discrepancies in patient, case, and procedure counts. All cases identified by sAReS as SSI candidates, regardless of their status in HygArzt, underwent targeted reassessment, whereas algorithm-negative cases were not systematically re-reviewed. Although all patients had previously undergone prospective SSI surveillance within the HygArzt study, some degree of verification bias cannot be excluded. sAReS-SSI still identifies too many *external* SSIs, as we deliberately included ambiguous and difficult cases as putative *in-house* SSIs, acknowledging that subsequent manual review was still necessary. Furthermore, our patient-based approach, which considers only one initial surgery and the first and last signs of an SSI, is a simplification. This approach may not fully capture the complex clinical pathways of patients undergoing multiple operations or experiencing multiple infection episodes. Consequently, it may not always be possible to attribute individual SSI events to specific procedures.

## Conclusion

Our newly developed algorithm, sAReS-SSI, identifies SSI candidates in routine hospital data with high accuracy and reduces false positives by 37.4% compared with NWIF. This demonstrates its potential to support efficient SSI surveillance and may support monitoring of procedures that are currently not included as indicator procedures in standard OP-KISS modules. By pre-selecting high-risk cases, algorithms such as sAReS-SSI can complement existing systems such as OP-KISS, enabling targeted expert review without replacing established surveillance methodologies or specialist assessment. Although our analysis focused on three wards specialising in orthopaedic and traumatological surgery within a single hospital, the underlying methodology may be adaptable to other surgical specialties and HAIs. However, further validation studies are needed before these applications can be recommended. In general, this approach provides a foundation for future studies and the expansion of semi-automated, retrospective surveillance to other HAIs.

## Supplementary Information

Below is the link to the electronic supplementary material.


Supplementary Material 1


## Data Availability

The datasets generated and/or analysed during the current study are not publicly available due to data protection. All data generated or analysed during this study is summarized in this published article and its supplementary information files.

## Abbreviations

sAReS-SSI: Semi-Automated Retrospective Surveillance of Surgical Site Infections
ASA: American Society of Anesthesiologists
CCI: Charlson Comorbidity Index
CIs: Confidence intervals
DRG: Diagnosis-related groups
EEA: European Economic Area
EU: European Union
HAIs: Healthcare-associated infections
HCWs: Healthcare workers
ICD-10: International Classification of Diseases, 10th edition
IPC: Infection prevention and control
IQTIG: Institut für Qualitätssicherung und Transparenz im Gesundheitswesen; Institute for Quality Assurance and Transparency in Healthcare
KISS: Krankenhaus-Infektions-Surveillance-System; hospital infection surveillance system
NPV: Negative predictive value
NWIF: Nosokomiale Wundinfektionen; case-related healthcare-associated wound infection
OP-KISS: Operative module of hospital infection surveillance system
OPS: Operationen- und Prozedurenschlüssel; surgery and procedure codes
PPV: Positive predictive value
RR: Relative risk
SSIs: Surgical site infections
