# Supplementary material for: Semi-Automated Retrospective Surveillance of Surgical Site Infections (sAReS-SSI) – using hospital routine data for SSI tracking
Source: Antimicrob Resist Infect Control. 2026 Aug 31;15:112. doi: 10.1186/s13756-026-01818-4 (PMC13531810; doi:10.1186/s13756-026-01818-4)
Supplement: Supplementary file 1 [file 13756_2026_1818_MOESM1_ESM.docx]

# Supporting Information

**Table S1. Covariates table: Patient, diagnosis, and procedure characteristics in pre- and post-intervention phases.** Covariates table including data from 2,298 patients. Comparison of characteristics in pre-intervention and post-intervention phases: age, gender, number of surgeries (OPS codes starting with 5-, any time and unique dates), top 10 initial surgeries, top 10 main diagnosis codes and CCI Quan Score with components for comorbidities. CCI, Charlson Comorbidity Index; ICD-10, International Classification of Diseases 10^th^ edition; OPS, surgery and procedure codes; SD; standard deviation. SSI, surgical site infection. *Age calculated based on age brackets from routine data. **CCI Quan Score based on the updated CCI version by Quan H et al. Am J Epidemiol 2011; 173: 676–682.

| Variable | OPS code/category | Pre-intervention  N=1,038 | Post-intervention  N=1,260 | p-value |
| --- | --- | --- | --- | --- |
| Age* (years (SD)) |  | 49.3 (20.0) | 49.1 (20.3) | 0.830 |
| Male gender |  | 585 (56.4%) | 716 (56.8%) | 0.822 |
| Female gender |  | 453 (43.6%) | 544 (43.2%) | 0.822 |
| CCI Quan Score** (mean (SD)) |  | 0.6 (1.3) | 0.5 (1.3) | 0.093 |
| Number of surgeries | | | |  |
| Number of valid OPS codes starting with 5- (performed any time) (mean (SD)) |  | 3.8 (3.4) | 3.8 (2.9) | 0.567 |
| Number of valid OPS codes starting with 5- (performed on unique dates) (mean (SD)) |  | 1.4 (1.0) | 1.3 (0.7) | <0.001 |
| Initial surgeries, top 10, three-digit OPS codes starting with 5- | | | |  |
| Repositioning of fracture and luxation | 5-79 | 256 (24.7%) | 360 (28.6%) | 0.035 |
| Arthroscopic joint operations | 5-81 | 219 (21.1%) | 309 (24.5%) | 0.052 |
| Endoprosthetic joint and bone replacement | 5-82 | 202 (19.5%) | 147 (11.7%) | <0.001 |
| Operations on other bones | 5-78 | 141 (13.6%) | 156 (12.4%) | 0.392 |
| Open surgical and other joint operations | 5-80 | 52 (5.0%) | 95 (7.5%) | 0.014 |
| Operations on muscles, tendons, fasciae, and bursae | 5-85 | 58 (5.6%) | 64 (5.1%) | 0.589 |
| Replantation, exarticulation, and amputation of extre-mities and other operations on the locomotor system | 5-86 | 26 (2.5%) | 26 (2.1%) | 0.479 |
| Surgical restoration and reconstruction of skin and hypodermis | 5-90 | 14 (1.3%) | 18 (1.4%) | 0.871 |
| Operations on the spine | 5-83 | 9 (0.9%) | 22 (1.7%) | 0.069 |
| Information about the graft and the materials used | 5-93 | 13 (1.3%) | 12 (1.0%) | 0.490 |

**Continuation of Table S1**

| Variable | OPS code/category | Pre-intervention  N=1,038 | Post-intervention  N=1,260 | p-value |
| --- | --- | --- | --- | --- |
| Initial main diagnosis, top 10, three-digit ICD-10 codes | | | |  |
| Fracture of lower leg, including ankle | S82 | 88 (8.5%) | 139 (11.0%) | 0.041 |
| Complications of internal orthopaedic prosthetic devices, implants or grafts | T84 | 106 (10.2%) | 94 (7.5%) | 0.020 |
| Dislocation, sprain, and strain of joints and ligaments of knee | S83 | 81 (7.8%) | 110 (8.7%) | 0.423 |
| Gonarthrosis [arthrosis of knee] | M17 | 80 (7.7%) | 79 (6.3%) | 0.177 |
| Fracture of femur | S72 | 75 (7.2%) | 77 (6.1%) | 0.285 |
| Fracture of shoulder and upper arm | S42 | 53 (5.1%) | 80 (6.3%) | 0.204 |
| Coxarthrosis [arthrosis of hip] | M16 | 59 (5.7%) | 69 (5.5%) | 0.829 |
| Fracture of forearm | S52 | 61 (5.9%) | 60 (4.8%) | 0.234 |
| Internal derangement of knee | M23 | 30 (2.9%) | 42 (3.3%) | 0.544 |
| Disorders of continuity of bone | M84 | 37 (3.6%) | 32 (2.5%) | 0.152 |
| Not KISS/KISS, top 10 | | | |  |
| Non-KISS procedures | Not_KISS | 459 (44.2%) | 568 (45.1%) | 0.680 |
| Arthroscopic surgeries | ART | 241 (23.2%) | 315 (25.0%) | 0.321 |
| Hip replacement in osteoarthritis | HPRO_A | 99 (9.5%) | 105 (8.3%) | 0.312 |
| Knee endoprothesis | KPRO | 84 (8.1%) | 85 (6.7%) | 0.218 |
| Surgeries on the upper ankle joint | OSG | 52 (5.0%) | 71 (5.6%) | 0.507 |
| Closed reduction of a fracture or physeal arrest with osteosynthesis | FPF_G | 29 (2.8%) | 38 (3.0%) | 0.753 |
| Spondylodesis | SPONDY | 15 (1.4%) | 27 (2.1%) | 0.214 |
| Hip replacement revision | HPRO_R | 18 (1.7%) | 17 (1.3%) | 0.453 |
| Shoulder endoprothesis | SPRO | 12 (1.2%) | 11 (0.9%) | 0.498 |
| Open Reduction and Internal Fixation (ORIF) of a proximal femur fracture | FPF_O | 10 (1.0%) | 7 (0.6%) | 0.256 |

**Continuation of Table S1**

| Variable | OPS code/category | Pre-intervention  N=1,038 | Post-intervention  N=1,260 | p-value |
| --- | --- | --- | --- | --- |
| CCI Quan Score | | | | |
| Overall (mean (SD)) |  | 0.6 (1.3) | 0.5 (1.3) | 0.093 |
| Mi – Myocardial infarction |  | 16 (1.5%) | 6 (0.5%) | 0.009 |
| Chf – Congestive heart failure |  | 70 (6.7%) | 62 (4.9%) | 0.062 |
| Pvd – Peripheral vascular disease |  | 69 (6.6%) | 67 (5.3%) | 0.179 |
| Cevd – Cerebrovascular disease |  | 37 (3.6%) | 36 (2.9%) | 0.336 |
| Dementia – Dementia |  | 28 (2.7%) | 29 (2.3%) | 0.544 |
| Cpd – Chronic pulmonary disease |  | 124 (11.9%) | 146 (11.6%) | 0.790 |
| Rheumd – Rheumatic disease |  | 17 (1.6%) | 19 (1.5%) | 0.803 |
| Pud – Peptic ulcer disease |  | 3 (0.3%) | 1 (0.1%) | 0.334 |
| Mld – Mild liver disease |  | 16 (1.5%) | 16 (1.3%) | 0.580 |
| Diab – Diabetes without chronic complications |  | 100 (9.6%) | 126 (10.0%) | 0.769 |
| Diabwc – Diabetes with chronic complications |  | 24 (2.3%) | 17 (1.3%) | 0.083 |
| Hp – Hemiplegia or paraplegia |  | 23 (2.2%) | 25 (2.0%) | 0.699 |
| Rend – Renal disease |  | 62 (6.0%) | 57 (4.5%) | 0.119 |
| Canc – Any malignancy, including lymphoma and leukemia, except malignant neoplasm of skin |  | 17 (1.6%) | 13 (1.0%) | 0.203 |
| Msld – Moderate or severe liver disease |  | 3 (0.3%) | 4 (0.3%) | 1.000 |
| Metacanc – Metastatic solid tumor |  | 6 (0.6%) | 8 (0.6%) | 0.862 |
| Aids |  | 0 (0.0%) | 0 (0.0%) | - |

**Table S2. Covariates table: Non-SSI vs. SSI patients (sAReS-SSI).** Covariates table including data from 2,298 patients. Comparison of patient characteristics in pre-intervention and post-intervention phases. AReS-SSI, semi-Automated Retrospective Surveillance of Surgical Site Infections; CCI, Charlson Comorbidity Index; SD, standard deviation; SSI, surgical site infection. *Age calculated based on age brackets from routine data. **Based on the updated CCI version by Quan H et al. Am J Epidemiol 2011; 173: 676–682.

| Variable | Pre-intervention | | | Post-intervention | | | | | |  |
| --- | --- | --- | --- | --- | --- | --- | --- | --- | --- | --- |
|  | **No SSI**  N=939 | **SSI**  N=99 | **p-value** | | **No SSI**  N=1,161 | | **SSI**  N=99 | | **p-value** | |
| Age* (years (SD)) | 49.0 (20.2) | 52.1 (17.5) | 0.102 | | 48.7 (20.3) | | 53.7 (19.2) | | 0.014 | |
| Male gender | 517 (55.1%) | 68 (68.7%) | 0.009 | | 654 (56.3%) | | 62 (62.6%) | | 0.225 | |
| Female gender | 422 (44.9%) | 31 (31.3%) | 0.009 | | 507 (43.7%) | | 37 (37.4%) | | 0.225 | |
| CCI Quan Score** (mean (SD)) | 0.5 (1.2) | 1.0 (1.7) | 0.007 | 0.4 (1.2) | | 0.9 (1.8) | | 0.005 | |  |

## Comparison of SSI vs. non-SSI patients, gender, and comorbidities (sAReS-SSI)

With respect to other patient characteristics (age, gender, comorbidities), SSI patients were older than non-SSI patients in both the pre-intervention (non-SSI vs. SSI: mean ± SD: 49.0 ± 20.2 vs. 52.1 ± 17.5 years; p = 0.102) and post-intervention phase, with a significant difference found only in the post-intervention phase (non-SSI vs. SSI: mean ± SD: 48.7 ± 20.3 vs. 53.7 ± 19.2 years; p = 0.014) (**Table S2**). In the pre-intervention phase, a higher proportion of males vs. females experienced an SSI (68.7% vs. 31.3%, p = 0.009), whereas this difference was not significant in the post-intervention phase (62.6% vs. 37.4%, p = 0.225) (**Table S2**). Semi-automated analysis using sAReS-SSI provided additional insights, including the calculation of the CCI Quan Score. SSI patients exhibited a significantly higher CCI Quan Score than non-SSI patients in the pre-intervention (non-SSI vs. SSI: mean ± SD: 0.5 ± 1.2 vs. 1.0 ± 1.7; p = 0.007) and the post-intervention phase (non-SSI vs. SSI: mean ± SD: 0.4 ± 1.2 vs. 0.9 ± 1.8; p = 0.005) (**Table S2**). When non-SSI and SSI patients were combined, the difference for the overall CCI Quan Score between the pre- and post-interventional phases was not significant (pre- vs. post-intervention: mean ± SD: 0.6 ± 1.3 vs. 0.5 ± 1.3; p = 0.093) (**Table S1**). A full list of the comorbidities relevant to the CCI Quan Score and their frequencies is also provided in **Table S1**, with chronic pulmonary disease being the most common in both the pre-intervention (n = 124; 11.9%) and post-intervention phase (n = 145; 11.6%).

Figure S1: Development of SSI rates over time. Numbers of patients with SSI and SSI rates according to sAReS-SSI classification during pre- (A) and post-intervention (B) phases of the HygArzt study. The average SSI rate was 9.5% in the pre-intervention phase and 7.9% in the post-intervention phase. AReS-SSI, semi-Automated Retrospective Surveillance of Surgical Site Infections; SMA, simple moving average; SSI, surgical site infection.
